# The cause and effect problem: Is there mutual obesity among Arab Israeli couples?

**DOI:** 10.1371/journal.pone.0240034

**Published:** 2020-10-16

**Authors:** Yuval Arbel, Chaim Fialkoff, Amichai Kerner

**Affiliations:** 1 Sir Harry Solomon School of Economics and Management, Western Galilee College, Acre, Israel; 2 Institute of Urban and Regional Studies, Hebrew University of Jerusalem, Mt. Scopus, Jerusalem; 3 School of Real Estate, Netanya Academic College, Netanya, Israel; University of Botswana, BOTSWANA

## Abstract

The influence of the health-related behavior of one spouse on that of the other is an important research question with public policy reprecussions. Yet, we are unaware of any previous study, which considered endogeneity problems between couples. Moreover, only a few studies considered ethnic origin differences among couples. Based on the 2016 wave of the Israeli longitudinal survey, we observe the cross-sectional correlation between the married couples' *BMI*, age, and accumulated wealth. The *BMI* (= $\frac{kg}{{meter}^{2}}$) is a conventional measure of obesity, where *BMI*≥25 is considered overweight. Using a 3SLS methodology (in an effort to correct the endogeneity problem associated with *BMI* couples), the analysis tests the mutual obesity hypothesis among married couples. This hypothesis states that the *BMI* of the male influences that of a female and vice versa. Results indicate that on the one hand, a one-percent *BMI* increase among Arab Israeli males is associated with a projected 0.969 percent BMI increase among Arab Israeli females (*p* = 0.017); and in the case that an Arab Israeli male suffers from overweight, the projected probability of his Arab Israeli female counterpart to suffer from overweight as well *rises* (*p* = 0.050). On the other hand, one cannot reject the null hypothesis that projected *BMI* of the Arab Israeli male is unaffected by that of his Arab Israeli female counterpart (*p* = 0.907 and *p* = 0.853). As for the Jewish Israeli population, in the case that the 3SLS methodology is employed, so that the endogeneity problem among couples is considered, a one-percent *BMI* increase among Jewish Israeli females is associated with a projected 0.639 percent *BMI* increase among Jewish Israeli males (*p* = 0.091). Unlike Arab Israeli couples, no support is found to indicate the influence in the other direction, namely, the *BMI* of the male influences that of the female spouse. Research findings may thus be of relevance to public health and policy planners. Two limitations of this research lie in: 1) the self-reported *BMI* (which might be different from the measured *BMI*); and 2) missing confounders, such as regional dummies, which are not available in the dataset.

## 1. Introduction

The influence of the health-related behavior of one spouse on that of the other is an important research question with public policy reprecussions. Yet, we are unaware of any previous study, which considered endogeneity problems between couples. Moreover, only a few studies have considered ethnic origin differences among couples (e.g., [1,2]).

In spite of the fact that overweight and obesity are known risk factors for a long list of health problems (e.g., [3–9]), no studies, to the best of our knowledge, have examined the impact of Body Mess Index (*BMI*) of the husband on that of the wife and vice versa while considering endogeneity problems. Endogenity is a prominent problem in data analysis (e.g., [10]). In particular, given the shared life and mutual management of a family, the *BMI* of the male potentially influence that of a female and vice versa. Overlooking endogenity problems in estimated equations often yields biased and inconsistent estimates (for methods to handle these problems, see, for example, [11]: 330–377; [12]: 305–317; [13]: 651–733).

The *BMI* measure equals $\frac{WEIGHT}{{HEIGHT}^{2}}=\frac{kg}{{meter}^{2}}$ (weight in kilograms divided by squared height in meters), and is considered a conventional measure for overweight (BMI≥25) and obesity (BMI≥30) [14]. The lack of physical activity and obesity have been identified by the World Health Organization (WHO) as a global pandemic and the fourth leading risk factor for global mortality responsible for an estimated 3.2–5.0 million deaths annually ([15,16]).

According to Cobb et al. [2], longitudinal studies to date regarding spouses have focused largely on white populations and have had limited follow-up or small sample sizes (e.g., [17–19]). Furthermore, previous studies have not accounted for potential confounding by individual characteristics, such as dietary intake, level of physical activity, and smoking status.

Among the few exceptions, Falba and Sindelar [20] examined changes over time in the health-related habits of each spouse as a function of changes in the health-related habits of the other spouse. Specifically, the authors analyzed changes in smoking, drinking, exercising, cholesterol screening, and obtaining a flu shot. Findings consistently showed that when one spouse improves his or her health-related behavior, the other spouse is likely to comply accordingly. This pattern is found across all the behaviors analyzed, and persists despite controlling for many other factors. Cobb *et*. *al*., [1] demonstrated that being married to a current smoker significantly decrease the odds of the female to quit smoking. Finally, Cobb *et*. *al*., [2] found that the condition of a female become obese nearly doubles the male spouse’s risk of becoming obese.

The current study seeks to extend the relatively small body of literature related to the health-related impact of married couples on each other in BMI terms, while considering ethnic origin and endogeneity problems. Following [21] and [10] and based on the 2016 wave of the Israeli longitudinal survey carried out by the Central Bureau of Statistics, we run a 3SLS simultaneous equation model separately for Jewish Israeli and Arab Israeli couples. The two endogenous variables are either the BMI of the wife and the husband, or BMI25, a dummy variable, which equals 1 for overweight, and 0 otherwise. The exogenous variables, which exactly identify the BMI equations of both the husband and the wife are the age of the spouse, namely, wife or husband, respectively. Finally, we incorporate a short series of socio-demographic exogenous variables at the family level (family size, car and home ownership, living in a detached single family home, and whether there is at least one book in the home library).

Results indicate that on the one hand, a one-percent *BMI* increase among Arab Israeli males is associated with a projected 0.969 percent *BMI* increase among Arab Israeli females (*p* = 0.017); and in the case that the Arab Israeli male suffers from overweight, the projected probability of his Arab Israeli female spouse to suffer from overweight as well *rises* (*p* = 0.050) On the other hand, one cannot reject the null hypothesis that projected *BMI* of the Arab Israeli male is unaffected by that of his Arab Israeli female spouse (*p* = 0.907 and *p* = 0.853).

As for the Jewish Israeli population, in the case that the 3SLS methodology is employed, so that the endogeneity problem among couples is considered, a one-percent *BMI* increase among Jewish Israeli females is associated with a projected 0.63875 percent *BMI* increase among Jewish Israeli males (*p* = 0.0909). Unlike Arab Israeli couples, no support is found to the influence in the other direction, namely, the *BMI* of the male influences that of a female. Research findings may thus be of assistance to public health and policy planners.

The rest of the article is organized as follows. Section 2 provides description of the data. Sections 3 and 4 describe the empirical model and results. Finally, section 5 summarizes and concludes with potential policy implications.

## 2. Description of the data

### 2a. Sample and controls

The data for this study are based on the 2016 wave of the longitudinal survey carried out by the Israeli CBS. As an OECD member since 2010, Israel is required to conduct such a survey.

None of the authors were involved directly or indirectly in the preparation of the survey, including sampling methods. The data were provided to us by representatives of the Israeli Social Science Data Center (ISDC) at the Hebrew University of Jerusalem, who were granted permission from the Israel Central Bureau of Statistics (ICBS). Moreover, it is generally accepted that surveys carried out by governmental agencies (e.g., the *PSID* in the United States) are based on rigorous sampling methods. In particular, individuals were sampled from the Israeli Population register. In addition, compared with private survey companies, government agencies have an important advantage; Individuals selected for the sample, are required by law to cooperate with the ICBS in the survey.

The survey sample is representative of the entire Israeli population, which includes all Israeli families and persons living in non-therapeutic institutions, namely, students in dormitories, assisted living for the elderly and absorption centers for new immigrants. The definition of the Israeli population excludes prisoners, inhabitants in therapeutic institutions, such as chronic care housing for the elderly, Israeli inhabitants who remained outside the country for at least one year, foreign workers, diplomats, and Bedouins living in scattered settlements in the southern part of Israel.

The common method used to collect the data from the sampled families is face-to-face interviews at the couple’s home using a computerized questionnaire. Interviews were held with each adult family member above 17 years old, in one of three languages (Hebrew, Arabic and Russian).

Given that several studies in the field use telephone interviews (e.g., [22,23]), the face-to-face interview technique provides two main advantages: 1) unlike phone interviews, the interviewee is more likely to answer the questionnaire in a non-threatening environment with a reduced tendency to avoid responding; 2) the interviewer can assess the reliability of answers, particularly regarding environmental housing conditions, and height and weight of each family member. 3) families who are sampled by the Israeli CBS are required to cooperate by law. Consequently, high response rates are obtained.

### 2b. Descriptive statistics

Given that the study examines mutual obesity among couples, the criteria to be included in the sample are: 1) couples (matched pairs); 2) availability of information regarding: a) weight and height of each couple from which the *BMI* is calculated; b) ethnic origin: Jewish or Arab; c) age of each spouse; d) family information referring to the variables incorporated in the regression analysis. Given the application of these criteria, the sample includes 1,950 matched pairs of males and females. The sample is stratified into 1,566 Jewish couples and 384 Arab couples, respectively.

Table 1 reports the descriptive statistics of the pooled sample. The table is divided according to personal characteristics, and family level variables, where the latter are identical for each couple. The sample consists of 3,900 individuals, who create 1,950 matched pairs of males and females. The sample is stratified into 1,566 Jewish couples and 384 Arab couples.

**Table 1:** Descriptive statistics: Pooled sample.

A. Personal Level							
	(1)	(2)	(3)	(4)	(5)	(6)	(7)
**VARIABLES Description**	**MALE Mean/Freq. (SD/proportion) Min-Max**	**FEMALE Mean/Freq. (SD/proportion) Min-Max**	**Actual Difference**^a^	**Actual Sample Size**	**Required Sample Size in the case that the actual difference equals the minimum required difference**	**Two sided alpha level for the required sample size**	**Method**
*WEIGHT_FE(MALE)* Weight in kg. wearing light clothing and without shoes	82.22 (13.50) 47–120	66.53 (12.27) 44–117	15.69***	1,950	11	5%	Pairwise t-test
*HEIGHT_FE(MALE)* Height in meters without shoes	1.75 (0.07) 1.52–1.90	1.63 (0.06) 1.49–1.87	0.12***	1,950	6	5%	Pairwise t-test
*BMI__FE(MALE)* $\frac{weight}{{height}^{2}}=\frac{kg}{{meter}^{2}}$	26.71 (4.03) 17.17–45.72	24.95 (4.50) 14.87–44.58	1.76***	1,950	73	5%	Pairwise t-test
*ln*(BMI*_FE(MALE*)) Natural logarithm of *BMI*	3.2739 (0.148)	3.2015 (0.1735)	0.0724***	1,950	62	5%	Pairwise t-test
*BMI*25*_FE(MALE)* 1 = overweight (*BMI≥*25), 0 = otherwise	1,171 (0.6005)	882 (0.4523)	(0.1482)***	1,950	148	5%	Pairwise t-test
*BMI*30*_FE(MALE)* 1 = type I obesity (*BMI≥*30), 0 = otherwise	361 (0.1851)	238 (0.1221)	(0.0630)***	1,950	443	5%	Pairwise t-test
*AGE_FE(MALE)*Age in years	49.25 (15.54) 21–80	46.03 (15.42) 19–80	3.22***	1,950	17	5%	Pairwise t-test
B. Family Level							
*ARAB* 1 = Arab Israeli; 0 = Jewish Israeli	384 (0.1969) 0–1	(0.18)	(0.0111)	1,950	4,143	5%	one-sample proportion test
*HHSIZE* Number of persons in the family	3.836 (1.819) 2–15	3.71	0.126**	1,950	1,638	5%	One-sample t test
*CAR* 1 = owner of at least one car; 0 = otherwise	1,591 (0.8159) 0–1	(0.703)	(0.1129)***	1,950	103	5%	one-sample proportion test
*BOOKS* 1 = has at least one book in the home library; 0 = otherwise	1,909 (0.9789) 0–1	N.A.	N.A.	1,950	N.A.	N.A.	N.A.
		*OWNER* 1 = owner of at least one apartment; 0 = otherwise	1,431 (0.7338) 0–1	1,950	476	5%	one-sample proportion test
*SINGLE_FAMILY* 1 = living in a detached single family unit; 0 = otherwise	571 (0.2928) 0–1	N.A.	N.A.	1,950	N.A.	N.A.	N.A.

^a^ Given lack of prior documentation, we assumed that the actual male-female difference equals the minimum required difference in a pre-study. ***p<0.001. Numbers in brackets at that column are the proportion differences.

^b^ The theoretical mean (if available) is obtained from ICBS reports at the national level. We assumed that the actual-theoretical difference equals the minimum required difference in a pre-study.

**p<0.005;

***p<0.001. Numbers in brackets at that column are the proportional differences.

As the descriptive statistics for the pooled sample indicate, the sample mean of the *WEIGHT* variable is 82.22 kg. for males and 66.53 kg for their female spouses. The 15.69 kg. gap is statistically different from zero at the 1‰ = 0.1% significance level. The sample mean of the *HEIGHT* variable is 1.75 meters for males and 1.63 meters for females. Once again, the 12 centimeters gap is statistically different from zero at the 1‰ = 0.1% significance level. Finally, the sample mean of the *BMI* variable is 26.71 for males and 24.96 for their female counterparts. The 1.76 points gap is statistically different from zero at the 1‰ = 0.1% significance level.

Analyzing the *BMI* formula for the sample mean, both the numerator and denominatior *rise* with the shift from females to their male spouses. The implication of the *BMI* increase with the same shift from females to males is the domination of the *WEIGHT* variable. Yet, one problem associated with this non-adjusted comparison may arise in the case that the male and female cohorts are different. Indeed, the sample mean of the *AGE* variable is 49.25 for males and 46.03 for females. The 3.22 year difference is statistically significant at the 1% significance level.

The family level variables include: *ARAB*, *HHSIZE*, *CAR*, *BOOKS*, *OWNER* and *SINGLE_FAMILY*. 19.69% $=\frac{384}{1,950}$ of the couples are Arab Israelis (*ARAB*). According to the Israeli national average $18\%=\frac{378}{2,100}$ of the families are Arabs ([24]: page 2). The average family size is 3.836 persons. The minimum family size is two persons, and the maximum is 15 persons (*HHSIZE*). The national average is 3.71 persons per family ([24]: page 2). 81.59% of the families own at least one car. The national Israeli average is 70.3% in 2016 ([25]: page 10) and 81.8% of the seventh income decile own at least one car ([25]: page 12). 97.89% of the families have at least one book in the home library (BOOKS). 73.38% of the families own at least one apartment (*OWNER*). The national Israeli average in 2017 is 67.6% (67.3% among Jewish families and 78.3% among Arab Israeli families [26]). Finally, 29.28% of the families live in a detached single-family housing unit (*SINGLE_FAMILY*).

## 3. Methodology

Consider the following simultaneous equations models applied separately to Jewish Israeli and Arab Israeli couples:

$$\text{ln}\;\left(BMI\_FEMALE\right)=a_{0}+a_{1}\;\text{ln}\left(BMI\_MALE\right)+a_{2}AGE\_MALE+a_{3}HHSIZE+a_{4}CAR+a_{5}BOOKS+a_{6}OWNER+a_{7}SINGLE\_FAMILY+u_{1}$$ (1)

$$\text{ln}\;\left(BMI\_MALE\right)=a_{0}^{'}+a_{1}^{'}\;\text{ln}\left(BMI\_FEMALE\right)+a_{2}^{'}AGE\_FEMALE+a_{3}^{'}HHSIZE+a_{4}^{'}CAR+a_{5}^{'}BOOKS+a_{6}^{'}OWNER+a_{7}^{'}SINGLE\_FAMILY+u_{1}^{'}$$ (2)

and

$$BMI25\_FEMALE=b_{0}+b_{1}BMI25\_MALE+b_{2}AGE\_MALE+b_{3}HHSIZE+b_{4}CAR+b_{5}BOOKS+b_{6}OWNER+b_{7}SINGLE\_FAMILY+u_{2}$$ (3)

$$BMI25\_MALE=b_{0}^{'}+b_{1}^{'}BMI25\_FEMALE+b_{2}^{'}AGE\_FEMALE+b_{3}^{'}HHSIZE+b_{4}^{'}CAR+b_{5}^{'}BOOKS+b_{6}^{'}OWNER+b_{7}^{'}SINGLE\_FAMILY+u_{2}^{'}$$ (4)

where ln(*BMI_FEMALE*) and ln(*BMI_MALE*), the endogenous variables in the structural system of Eqs (1) and (2), are the natural logarithms of the *BMI* of the female and male, respectively; *BMI25_FEMALE* and *BMI25_MALE*, the endogenous variables in the structural system of Eqs (3) and (4) are dummy variables that equals 1 if the female or male suffers from overweight, namely, *BMI*≥25, and 0 otherwise. *AGE_FEMALE* and *AGE_MALE* are the age of the female and male, and are considered the exogenous variables, which exactly identify the male and female equations, respectively; *HHSIZE* is the family size; *CAR*, *BOOKS*, *OWNER*, *SINGLE_FAMILY* are control dummy variables that equals 1 if the family owns at least one car, has at least one book in the home library, owns at least one apartment, lives in a single-family unit, and zero otherwise; *a*_0_, *a*_1_, *a*_2_, ⋯*a*_7_,; $a_{0}^{'},a_{1}^{'},a_{2}^{'},\cdots ,a_{7}^{'};$ *b*_0_, *b*_1_, *b*_2_, ⋯*b*_7_, $b_{0}^{'},b_{1}^{'},b_{2}^{'},\cdots ,b_{7}^{'}$ are parameters, and $u_{1},u_{1}^{'},u_{2},u_{2}^{'}$ are the stochastic random disturbance terms.

A prerequisite to address the endogeneity problem is the identification of each equation. The implication is that each equation includes at least one exogenous variable, which is omitted from the other equation. In the current example, the age of the spouse provides the exogenous variable, which identifies the other equation.

Age is known to be an excellent exogenous variable (the time variable is the only one that influences age). Other exogenous confounders are considered conventional demographic and socio-economic variables and were chosen based on availibity of data.

One method to address the endogeneity problem is the 3SLS consisting of three steps: 1) solving the model so that the model is formulated in reduced form; 2) calculating projected values from the reduced form equations, while adjusting them to become instrumental variables; 3) replacing the original endogenous variables at the right-hand side of each structural equation with the projected values obtained from the reduced form equation. In the current example, after substitution and rearranging terms, the reduced form equations are given by:

$$\text{ln}\;\left(BMI\_FEMALE\right)=c_{0}+c_{1}AGE\_FEMALE+c_{2}AGE\_MALE+c_{3}HHSIZE+c_{4}CAR+c_{5}BOOKS+c_{6}OWNER+c_{7}SINGLE\_FAMILY+e_{1}$$ (5)

$$\text{ln}\;\left(BMI\_MALE\right)=c_{0}^{'}+c_{1}^{'}AGE\_FEMALE+c_{2}^{'}AGE\_MALE+c_{3}^{'}HHSIZE+c_{4}^{'}CAR+c_{5}^{'}BOOKS+c_{6}^{'}OWNER+c_{7}^{'}SINGLE\_FAMILY+e_{1}^{'}$$ (6)

And

$$BMI25\_FEMALE=d_{0}+d_{1}AGE\_FEMALE+d_{2}AGE\_MALE+d_{3}HHSIZE+d_{4}CAR+d_{5}BOOKS+d_{6}OWNER+d_{7}SINGLE\_FAMILY+e_{2}$$ (7)

$$BMI25\_MALE=d_{0}^{'}+d_{1}^{'}AGE\_FEMALE+d_{2}^{'}AGE\_MALE+d_{3}^{'}HHSIZE+d_{4}^{'}CAR+d_{5}^{'}BOOKS+d_{6}^{'}OWNER+d_{7}^{'}SINGLE\_FAMILY+e_{2}^{'}$$ (8)

Application of the 3SLS methodology yields consistent estimates. The implication is that if the sample size approaches infinity, the estimated parameters equal those in the population.

## 4. Results

To carry out the analysis, the Stata 16 software package was employed (replication instructions of the outcomes are given in S1A Appendix). Table 2 reports the regression outcomes obtained from the 3SLS methodology. According to the table, on the one hand, there is no evidence that the female BMI is influenced by that of her male BMI counterpart (*p =* 0.483). On the other hand, a one percent *BMI* rise among the Jewish Israeli females is associated a projected 0.639 percent *BMI* increase among their Jewish Israeli male spouse (*p* = 0.091). Referring to the Arab Israeli couples, a one-percent *BMI* increase among Arab Israeli males is associated with a projected 0.969 percent *BMI* increase among their Arab Israeli female spouse (*p* = 0.017); and in the case that Arab Israeli male suffers from overweight, the projected probability of his Arab Israeli female counterpart to suffer from overweight as well *rises* (*p* = 0.050). Unlike the Jewish Israeli couples, there is no evidence that the *BMI* of the female influences that of the BMI of the male spouse among Arab Israeli couples (*p* = 0.907).

**Table 2:** 3SLS simultaneous equation model: Jewish Israeli vs. Arab Israeli couples^a^.

	(1)	(2)	(3)	(4)	(5)	(6)	(7)	(8)
VARIABLES	ln(BMI_FEMALE)	ln(BMI_MALE)	ln(BMI_FEMALE)	ln(BMI_MALE)	BMI25_FEMALE	BMI25_MALE	BMI25_FEMALE	BMI25_MALE
Constant	-3.193	1.246	-0.155	-54.952	-0.661	0.044	-0.77059**	2.39377
	(0.718)	(0.274)	(0.906)	(0.912)	(0.544)	(0.938)	(0.021)	(0.819)
Proj[ln(BMI_MALE)]	1.958	–	0.969**	–	–	–	–	–
	(0.483)	–	(0.017)	–	–	–	–	–
Proj[ln(BMI_FEMALE)]	–	0.639*	–	19.366	–	–	–	–
	–	(0.090)	–	(0.907)	–	–	–	–
Proj[BMI25_MALE]	–	–	–	–	3.054	–	1.093*	–
	–	–	–	–	(0.390)	–	(0.050)	–
Proj[BMI25_FEMALE]	–	–	–	–	–	2.333	–	10.431
	–	–	–	–	–	(0.539)	–	(0.852)
AGE_MALE	-0.001	–	0.004***	–	-0.011	–	0.010***	–
	(0.919)	–	(<0.001)	–	(0.639)	–	(<0.001)	–
AGE_FEMALE	–	-0.0002	–	-0.107	–	-0.016	–	-0.144
	–	(0.910)	–	(0.908)	–	(0.666)	–	(0.857)
HHSIZE	-0.001	-0.0003	0.015***	-0.228	-0.027	-0.0347	0.041**	-0.436
	(0.919)	(0.940)	(0.008)	(0.905)	(0.656)	(0.676)	(0.017)	(0.853)
CAR	-0.057*	0.0344*	-0.054*	0.395	-0.183	0.301	-0.086	-0.120
	(0.065)	(0.070)	(0.057)	(0.898)	(0.150)	(0.516)	(0.357)	(0.925)
BOOKS	0.074	-0.038	-0.00525	0.450	0.150	0.154	0.010	0.283
	(0.644)	(0.531)	(0.894)	(0.911)	(0.818)	(0.787)	(0.936)	(0.882)
OWNER	-0.001	0.003	0.016	-0.046	-0.018	0.004	0.035	-0.048
	(0.760)	(0.739)	(0.609)	(0.931)	(0.854)	(0.957)	(0.718)	(0.945)
SINGLE_FAMILY	-0.004	0.003	-0.00683	-0.306	-0.039	0.113	-0.080	0.214
	(0.810)	(0.755)	(0.811)	(0.914)	(0.640)	(0.569)	(0.379)	(0.840)
Ethnic Group	JEWISH	JEWISH	ARAB	ARAB	JEWISH	JEWISH	ARAB	ARAB
Observations	1,566	1,566	384	384	1,566	1,566	384	384
Chi_SQ (df = 7)	63.87***	73.81***	54.49***	0.0559	16.47**	12.23*	36.50***	0.123
	(<0.001)	(<0.001)	(<0.001)	(0.999)	(0.021)	(0.093)	(<0.001)	(0.999)

^a^ We use the 3SLS methodology to test the mutual obesity hypothesis. Columns (1)-(2) and (3)-(4) [(5)-(6) and (7)-(8)] are the structural equations given by (1) and (2) [(3) and (4)]–after replacement by the projected values obtained from the reduced-form equations–and applied separately to Jewish and Arab couples. P-values are given in parentheses.

***p<0.01,

**p<0.05,

*p<0.1.

Referring to the control variables, the projected probability of a female Arab Israeli *BMI* rises by 0.4% following a one-year increase in the age of her Arab male counterpart (*p*<0.001), and by 1.5% following an increase in the family’s size by one person (*p* = 0.008).

## 5. Discussion

Previous literature has identified obesity and overweight as the fourth leading risk factor for global mortality, responsible for an estimated 3.2–5.0 million deaths annually (e.g., [15,16,27]). Recently, Nyberg *et*. *al*. [28] estimated the loss of disease-free years associated with class II–III obesity (*BMI*≥35, *BMI* = *WEIGHT*÷(*HEIGHT*^2^), where *WEIGHT* is measured in kilograms and *HEIGHT* is measured in meters) to range between 7.1 and 10.0 years in subgroups of participants with different socioeconomic status, levels of physical activity, and smoking habits.

Based on the 2016 wave of the Israeli longitudinal survey, we observe the cross-sectional correlation between the *BMI*, age, and accumulated wealth of the married couple. Using the 3SLS methodology (in an effort to correct the endogeneity problem associated with *BMI* couples, and thus prevent a potential bias and generate consistent estimates), the analysis tests the mutual obesity hypothesis among married couples (for the 3SLS methodology, see, for example, [12]: 305–317; [13]: 651–733; [5]: 330–377). This hypothesis states that the *BMI* of the male influences that of a female and vice versa. Finally, we apply the model separately based on ethnic origin.

Results indicate that on the one hand, a one-percent BMI increase among Arab Israeli males is associated with a projected 0.969 percent BMI increase among Arab Israeli females (*p* = 0.017); and in the case that Arab Israeli male suffers from overweight, the projected probability of his Arab Israeli female spouse to suffer from overweight as well *rises* (*p* = 0.05004). On the other hand, one cannot reject the null hypothesis that projected *BMI* of the Arab Israeli male is unaffected by that of his Arab Israeli female counterpart (*p* = 0.9066 and *p* = 0.8525). As for the Jewish Israeli population, in the case that the 3SLS methodology is employed, so that the endogeneity problem among couples is considered, a one-percent *BMI* increase among Jewish Israeli females is associated with a projected 0.63875 percent *BMI* increase among Jewish Israeli males (*p* = 0.0909). Unlike Arab Israeli couples, no support is found to the influence in the other direction, namely, the *BMI* of the male influences that of a female. Research findings may thus be of assistance to public health and policy planners.

Two limitations of this research lie in: 1) the self-reported *BMI* (which might be different from the measured *BMI*); and 2) missing confounders, such as regional dummies, which are not available in the dataset.

Referring to the self-reported *BMI*, S1B Appendix exhibits Overweight or Obese Population (Measured/Self Reported, % of Population Aged 15+, 2018 or Latest Available) in OECD countries [29]. As the figure demonstrates, unlike many OECD countries (e.g., Luxenburg, Australia, Chile), the gap between the measured and self-reported *BMI* in Israel is rather small. Both measures indicate that around 50% of the Israeli population suffer from overweight and obesity.

Referring to missing confounders, such as regional dummies, rural or urban surroundings may potentially affect the results. It is anticipated that the mutual obesity among couples will be different based on occupation (e.g., agriculture vs. office services) and living area (rural vs. urban). Obviously, this is a subject for future research.

## Supporting information

S1 File (ZIP)

S1 Appendix (DOCX)
