# Supplementary material for: The cause and effect problem: Is there mutual obesity among Arab Israeli couples?
Source: PLoS One. 2020 Oct 16;15(10):e0240034. doi: 10.1371/journal.pone.0240034 (PMC7567363; doi:10.1371/journal.pone.0240034)
Supplement: S1 Appendix — (DOCX) [file pone.0240034.s002.docx]

**8. Appendix A: Replication Instructions**

For replication objectives, the following Stata 16 files are attached:

1. Raw Data Files:

| Number | Year of Interview | File Name | Description |
| --- | --- | --- | --- |
| **S1** | 2016 | “S1.f805fam.dta” | Raw family level data file obtained from a representative sample of the same families interviewed in 2016 |
| **S2** | 2016 | “S2.f805ind.dta” | Raw individual level data file obtained from a representative sample of the same families interviewed in 2016 |

1. Other files

| Number | Type of File | File  Name | Description |
| --- | --- | --- | --- |
| **S3** | Batch File | “S3.For_PLOS_ONE_20200719.do” | A batch file designed to replicate the results in Stata software package |

Replication is possible by running the batch files given by S3, after updating the change directory “cd” command in the first row, so as to fit the user PC directory, where the raw files are stored and downloaded.

The following Table provides the names of the variables in the text of the article and the programming code

| **VARIABLES**  **Description** | **Programming Code** |
| --- | --- |
| *WEIGHT_FE(MALE)*  Weight in kg. wearing light clothing and without shoes | Indweigh0 (for male)  Indweigh1 (for female) |
| *HEIGHT_FE(MALE)*  Height in meters without shoes | height0 (for male)  height1 (for female) |
| *BMI__FE(MALE)*  $\frac{\boldsymbol{weight}}{\boldsymbol{height}^{\boldsymbol{2}}}\boldsymbol{=}\frac{\boldsymbol{kg}}{\boldsymbol{meter}^{\boldsymbol{2}}}$ | bmi0 (for male)  bmi1 (for female) |
| *ln*(*BMI_FE(MALE*))  Natural logarithm of *BMI* | lbmi0 (for male)  lbmi1 (for female) |
|  |  |
| *BMI*25*_FE(MALE)*  1=overweight (*BMI≥*25), 0=otherwise | bmi25_0 (for male)  bmi25_1 (for female) |
| *BMI*30*_FE(MALE)*  1=type I obesity (*BMI≥*30), 0=otherwise | bmi30_0 (for male)  bmi30_1 (for female) |
| *AGE_FE(MALE)*  Age in years | age_0 (for male)  age_1 (for female) |

**9. Appendix B: Overweight or Obese Population (Measured/Self Reported, % of Population Aged 15+, 2018 or Latest Available)**

**
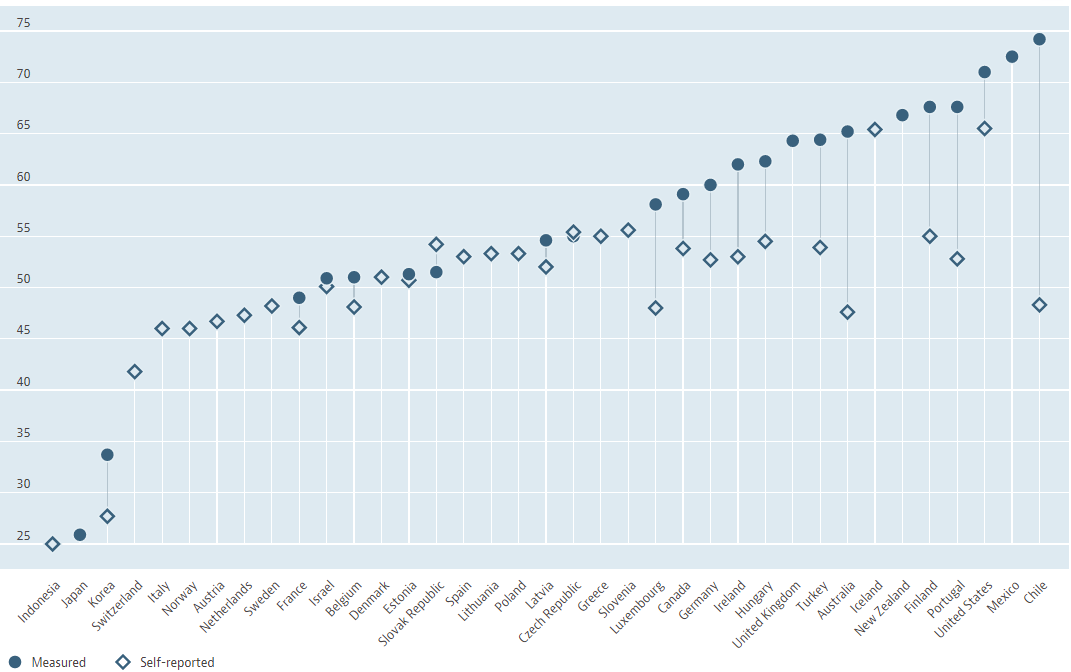
**

Source : OECD Report available at: <https://data.oecd.org/pinboard-editor/> . Overweight is defined as: $25\leq BMI<30$ and obesity is defined as $BMI\geq30$, where $BMI=\frac{kg}{{meter}^{2}}$.
